# Exploring the Reproductive Toxicity of Bisphenol S Through a Network Toxicology and Molecular Docking Analysis

**DOI:** 10.1002/pdi3.70036

**Published:** 2026-04-12

**Authors:** Siyuan Wang, Yan Fu, Xu Huang, Qitong Guo, Xiangqin Zheng, Shengde Wu, Lianju Shen, Guanghui Wei

**Affiliations:** ^1^ Department of Urology Children's Hospital of Chongqing Medical University Chongqing China; ^2^ Chongqing Key Laboratory of Structural Birth Defect and Reconstruction, Ministry of Education Key Laboratory of Child Development and Disorders Pediatric Research Institute National Clinical Research Center for Child Health and Disorders Children's Hospital of Chongqing Medical University Chongqing China

**Keywords:** BPS, molecular docking, network toxicology, reproductive injury

## Abstract

The aim of this study was to investigate the potential reproductive toxicity of bisphenol S (BPS) and the related molecular mechanisms through a network toxicology approach. By utilizing various databases, including the Comparative Toxicogenomics Database (CTD), GeneCards, Online Mendelian Inheritance in Man (OMIM), the Pharmacogenomics Knowledgebase (PharmGKB), and the Therapeutics Target Database (TTD), and limiting the species to *Homo sapiens*, we confirmed 45 potential targets related to both BPS exposure and reproductive injury. Additional analysis via STRING and Cytoscape pinpointed 4 key targets: AKT serine/threonine kinase 1 (AKT1), interleukin‐6 (IL‐6), interleukin‐1‐beta (IL‐1β), and tumor necrosis factor α (TNFα). Gene Ontology (GO) and Kyoto Encyclopedia of Genes and Genomes (KEGG) pathway analyses indicated that the principal targets of BPS‐mediated reproductive damage are mainly involved in oxidative stress signaling and cell secretion pathways. Because PI3K/AKT regulates the secretion of IL‐6, IL‐1β, and TNFα, we focus on AKT1 in the molecular docking experiments. Unexpectedly, we found a strong interaction between BPS and AKT1. Furthermore, the results suggested that women may be more susceptible to BPS‐mediated reproductive toxicity than men. This study provides a theoretical framework for understanding the molecular mechanisms through which BPS causes reproductive toxicity and lays the foundation for preventing and treating reproductive disorders related to BPS exposure. In addition, our network toxicology‐based method can accelerate the discovery of pathways involved in the toxic effects of environmental chemicals that are currently unknown.

## Graphical abstract

Utilizing network toxicology, molecular docking, and dynamic simulations to investigate the potential reproductive toxicity of bisphenol S (BPS).

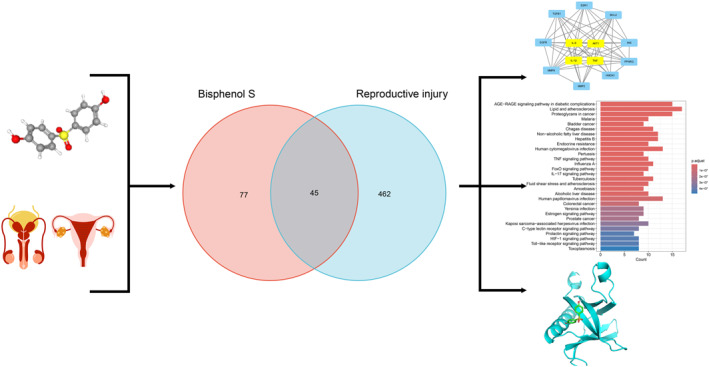

## 1 Introduction

Bisphenol A (BPA) is a prevalent synthetic chemical often used in making various plastic items such as eyeglasses, packaging, toys, plastic containers, and other daily products [1]. BPA has attracted considerable interest because of its strong ability to disrupt endocrine function, especially its harmful impact on reproductive health [2]. Increasing public apprehension about BPA has prompted a transition to “BPA‐free” products, which often include bisphenol analogs, notably bisphenol S (BPS) [3, 4]. As the primary substitute for BPA, BPS is used in many industrial applications and consumer products, such as personal care products, food packaging, clothing, synthetic polymers, and thermal paper [5]. Then it enters the human body through a variety of direct and indirect ways, such as skin contact, oral ingestion, or breathing [6], and leads to toxic effects on human health [7].

Exposure to BPS during pregnancy and breastfeeding can lead to various issues in offspring, including restricted fetal growth, delayed neurodevelopment, and metabolic disturbances [8]. Moreover, the risk of miscarriage and premature birth may be linked to BPS exposure, although concrete evidence remains limited. Meanwhile, numerous epidemiological studies have detected BPS in many urine samples, with concentrations ranging from 0.03 μg·L^−1^ to 0.4 μg·L^−1^ [9]. At these exposure levels, BPS has been found to be correlated with the development of obesity and diabetes, potentially resulting in metabolic disorders. In addition, BPS can also lead to, for example, intestinal microenvironment alterations and nervous system abnormalities [10]. Moreover, BPS exposure may increase the risk of cancer [11]. It is worth noting that BPS, as an environmental endocrine disruptor, has been frequently reported to cause reproductive toxic damage.

For female reproductive impairment, BPS exposure reduced the number of ovarian granulosa cells [12], decreased occyte quality and ovarian reserve function [13, 14], and its exposure in pregnancy also resulted in transgenerational toxicity [15]. For the adverse effects on male reproductive function, BPS exposure leads to blood‐testis barrier disruption [16], impairment of testosterone synthesis [17], testicular inflammation [18], and spermatogenesis dysfunction [19].

Reproduction is essential for species survival as it ensures the continuation of life, involving complex physiological changes. The occurrence of sexual reproduction requires the participation of diverse cell types, a spectrum of hormones, and sophisticated signaling pathways (including the AMPK pathway and the mTOR pathway) [20]. Yet, human fertility is increasingly fragile. It is abnormally sensitive to toxicants, especially to environmental endocrine disruptors. However, the impact of BPS on reproductive health can occur through various mechanisms, making it essential to determine specific targets for the precise treatment of the reproductive harm it causes.

In this study, we utilized public databases, network toxicology, and molecular docking analysis to predict the targets of BPS‐induced reproductive damage, aiming to provide a scientific basis for subsequent mechanistic studies and precise treatment.

## 2 Methods

### 2.1 Identification of Potential BPS Targets

Potential targets of BPS were identified through a search for “bisphenol S” in the Comparative Toxicogenomics Database (CTD) [21]. To establish a robust correlation between the potential genes and BPS, we filtered the genes with an interaction count of five or more in order to create the target library for BPS.

### 2.2 Selection of Targets Related to Reproductive Toxicity

To pinpoint potential reproductive toxicity‐related targets, a thorough search was carried out on a global scale across various databases, including GeneCards, Online Mendelian Inheritance in Man (OMIM), the Pharmacogenomics Knowledgebase (PharmGKB), and the Therapeutics Target Database (TTD), limiting the species to *Homo sapiens*, and utilizing search terms such as “reproductive harm” and “reproductive toxicity” [22]. The primary focus was on genes that were strongly associated with reproductive injury and toxic effects, with an emphasis on genes with a GeneCards database score equal to or greater than 10. The median score was established as the threshold, leading to the selection of genes with higher scores for the creation of libraries specifically targeting reproductive toxicity. Venn diagrams were subsequently used to identify shared BPS and reproductive injury‐related targets, highlighting these shared targets as potential contributors to the reproductive toxicity induced by BPS.

### 2.3 Construction of Protein–Protein Interaction (PPI) Networks and Screening of Core Targets

The targets associated with BPS and reproductive toxicity, identified through Venn diagrams, were submitted to the STRING database [23]. The selection of species was initially limited to *Homo sapiens* in order to focus on the active reproductive injury's target proteins corresponding to the target genes. The “minimum required interaction score” was set to a “high confidence” level of 0.7, and to clarify the interactions of potential targets, the option to “hide disconnected nodes in the network” was activated. Utilizing data from the STRING database, Cytoscape software (version 3.10.2) was employed to analyze the characteristics of individual nodes and visualize the molecular interactions [24]. This process resulted in the generation of a PPI network diagram, enabling the evaluation of the structural properties of both nodes and edges within the network. The primary targets related to reproductive toxicity caused by BPS were identified based on the following criteria: ① betweenness centrality exceeding the median, ② closeness centrality exceeding the median, ③ degree centrality exceeding the median, ④ eigenvector centrality exceeding the median, ⑤ local average connectivity centrality exceeding the median, and ⑥ network degree centrality exceeding the median. Additionally, the central gene in the PPI network was validated using the CytoNCA plug‐in.

### 2.4 Functional Pathway Enrichment Analyses

In order to functionally annotate the potential targets linked to reproductive damage induced by BPS, Gene Ontology (GO) and Kyoto Encyclopedia of Genes and Genomes (KEGG) pathway enrichment analyses were conducted using the DAVID and FUMA webtools [25, 26]. This research identified the biological process (BP), cellular component (CC), and molecular function (MF) terms within the shared targets that showed significant enrichment. Additionally, pathways enriched in the possible targets related to BPS‐mediated reproductive impairment were revealed through KEGG enrichment analysis, employing a false discovery rate (FDR) threshold of less than 0.05 to highlight the primary pathways involved in the toxic effects of BPS [27, 28]. To effectively present the results from both GO and KEGG analyses, RStudio was employed for visualizing the outcomes.

### 2.5 Molecular Docking of BPS With Core Targets

To gain a more comprehensive understanding of the molecular interactions between BPS and the primary target proteins, molecular docking techniques were employed to model the engagement of BPS with the core proteins identified in this research. The 2D representation of BPS was sourced from PubChem and subsequently imported into Chem3D to derive its 3D structure. The crystal structures of the core proteins were acquired from the RCSB Protein Data Bank (PDB) [29]. PyMOL facilitated the removal of the original ligand and water molecules from the selected proteins, which were then processed in AutoDock Tools 1.5.7 for hydrogenation, charge calculations, and the adjustment of nonpolar hydrogen binding. Prior to executing AutoDock Vina using command line operations, the parameters for the periodic box size and genetic algorithm were established. The final phase encompassed visualizing the findings using Discovery Studio and PyMOL.

### 2.6 Molecular Dynamics of BPS With Core Targets

Gromacs 2022.3 software was used for molecular dynamics simulation [30]. For the preprocessing of small molecules, AmberTools 22 was utilized to apply a GAFF force field to these molecules, whereas Gaussian 16W facilitated the hydrogenation of the small molecules and enabled the calculation of the restrained electrostatic potential (RESP). The potential data generated were incorporated into the topology file of the molecular dynamics system. The simulation was conducted under static conditions at a temperature of 300 K and a pressure of 1 bar. The force field employed was Amber99sb‐ildn, and water molecules, modeled using the Tip3p water model, served as the solvent. To ensure the charge neutrality of the simulation system, an appropriate quantity of Na^+^ ions was added. The steepest descent method was implemented for energy minimization, followed by isothermal isovolumetric ensemble equilibrium and isothermal isobaric ensemble equilibrium, with a total of 100,000 steps, a coupling constant of 0.1 ps, and a duration of 100 ps. Subsequently, a free molecular dynamics simulation was conducted, comprising 5,000,000 steps with a timestep of 2 fs, culminating in a total duration of 100 ns. Upon the completion of the simulation, the analysis tools included within the software were employed to assess the trajectory and compute parameters such as root‐mean‐square deviation (RMSD), root‐mean‐square fluctuation (RMSF), radius of gyration (GYRATE), solvent‐accessible surface area (SASA), formation of hydrogen bonds (H‐Bonds), and the Gibbs energy landscape.

## 3 Results

### 3.1 Preliminary Network Toxicity Analysis of BPS

After the database search results were analyzed, we obtained a basic understanding of the reproductive toxicity caused by BPS. Toxicity models suggest that BPS may cause reproductive damage through oxidative stress and alterations in cell secretion function, which is consistent with several previous studies on reproductive toxicity caused by BPS. These findings lay the foundation for further exploration of the toxic effects of BPS on the human body.

### 3.2 Identification of Potential Reproductive Injury‐Targets of BPS

**FIGURE 1:**
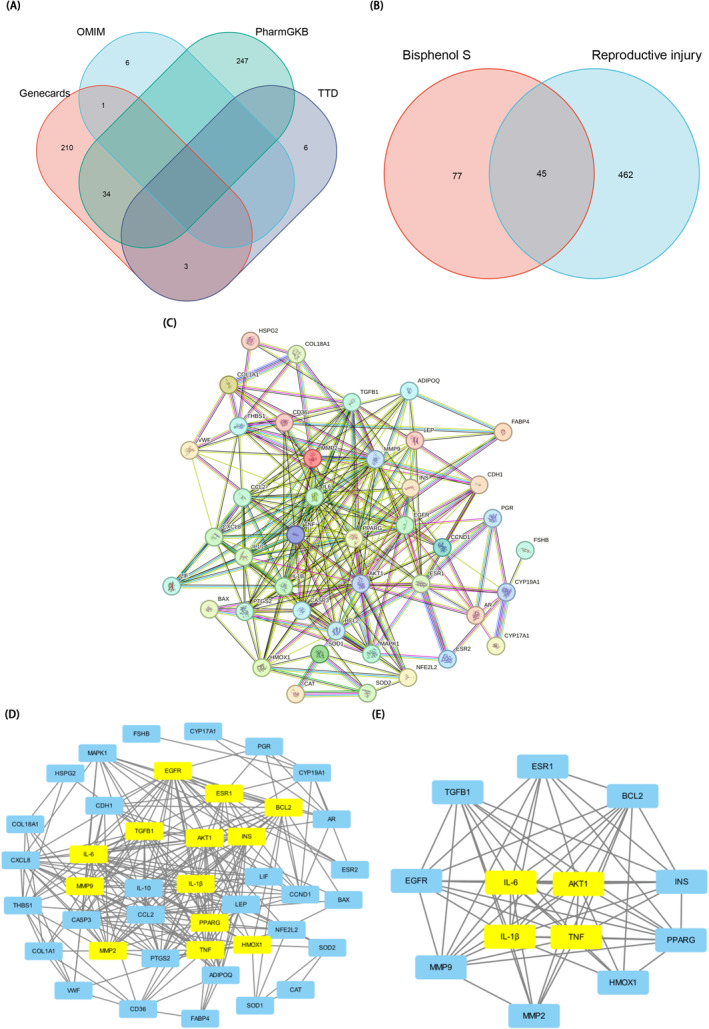
Core genes of BPS targets and reproductive injury‐related targets. (A) Reproductive injury‐related targets. (B) Shared BPS and reproductive injury‐related targets. (C) The PPI network of potential targets. (D) The PPI network of core targets. (E) Targets strongly associated with reproductive toxicity and BPS. BPS, bisphenol S; PPI, protein–protein interaction.

First, we identified 122 BPS targets using the CTD databases. We then identified 507 targets strongly associated with reproductive damage using GeneCards, OMIM, PharmGKB, and the TTD (Figure 1A). Merging these sets and eliminating duplicates resulted in 45 overlapping targets, which were identified as potential targets for BPS‐mediated reproductive injury (Figure 1B).

### 3.3 Construction of a PPI Network and Extraction of Core Genes

By importing the 45 identified targets into the STRING database and filtering out isolated targets, we established a PPI network with 42 nodes and 255 edges (Figure 1C). Using Cytoscape software, we analyzed the topological properties of the network nodes, focusing on their degree and betweenness centrality. Additionally, Cytoscape software was used to visualize the PPI network.

Upon conducting the network analysis, we identified the core targets responsible for BPS‐mediated reproductive toxicity on the basis of their degree values (Table 1). To illustrate the interactions among these core targets, we created a diagram (Figure 1D). The core targets are highlighted with a yellow background, with the top four being IL‐6 (interleukin‐6), IL‐1β (interleukin‐1‐beta), AKT1 (AKT serine/threonine kinase 1), and TNFα (tumor necrosis factor) (Figure 1E). Previous studies have validated the crucial roles of proteins encoded by these genes in various cellular functions, such as immunity, metabolism, and inflammatory responses.

**TABLE 1:** Key targets identified from protein–protein interaction networks.

Name	Betweenness	Closeness	Degree	Eigenvector	Local average connectivity (LAC)	Network degree
TGFB1	1.29127	0.8125	10	0.25709	8	9.079365
AKT1	4.880952	1	13	0.312795	9.384615	13
MMP2	0.222222	0.722222	8	0.213473	6.75	7.714286
IL‐6	4.880952	1	13	0.312795	9.384615	13
HMOX1	0.444444	0.684211	7	0.186689	5.428571	6.333333
ESR1	0.422222	0.722222	8	0.208859	6.5	7.428571
EGFR	1.688095	0.866667	11	0.279042	8.727273	10.17778
MMP9	2.471429	0.866667	11	0.275582	8.363636	9.92381
INS	1.488095	0.8125	10	0.255712	7.8	8.857143
TNF	3.259524	0.928571	12	0.295313	9	11.47778
BCL2	3.261905	0.866667	11	0.269786	8	9.725397
PPARG	2.32619	0.8125	10	0.248834	7.4	8.634921
CASP3	2.481746	0.866667	11	0.274273	8.363636	10
IL‐1β	4.880952	1	13	0.312795	9.384615	13

### 3.4 Functional and Pathway Enrichment Analyses of Potential Targets

**FIGURE 2:**
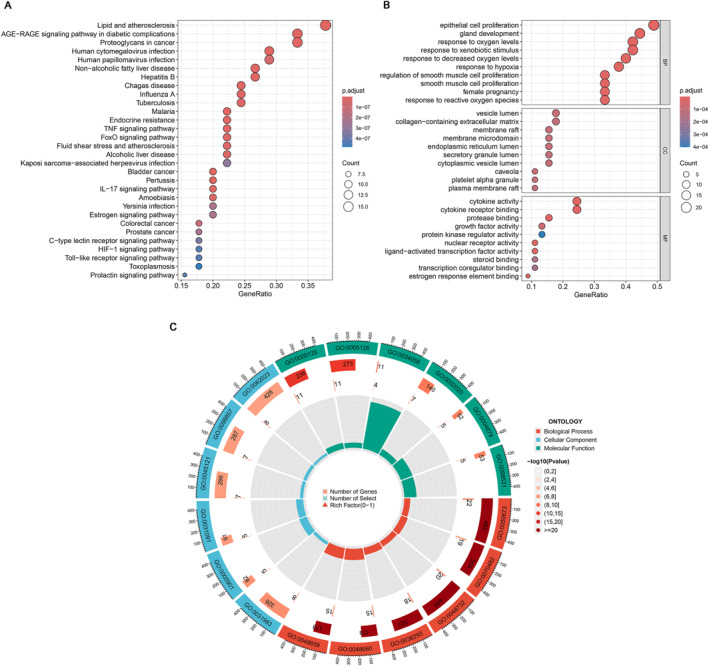
KEGG and GO enrichment analyses of putative targets. (A) Bubble chart displayed the top 20 enriched KEGG pathways in descending order of their FDR values. Each bubble represents a specific pathway, with its size corresponding to the number of genes enriched in that pathway. The color intensity of the bubbles signifies the importance of the enrichment, with darker red indicating greater significance. (B) Bubble diagrams were generated to present the top 10 GO enriched terms for each category—BPs, CCs, and MFs—with decreased FDR values among the 42 potential targets. A lower FDR value represents greater statistical significance. The size of the bubbles indicates the degree of gene expressions within a particular pathway, whereas the color saturation reflects the level of enrichment. These enriched terms offer valuable insights into crucial BPs, CCs, and MFs that could be impacted by exposure to BPS. (C) The circular plot illustrates the significant biological processes, cellular components, and molecular functions identified through GO enrichment analysis. Each sector represents a specific GO term, with the depth of color indicating the level of enrichment significance; a higher value reflects greater significance. BP, biological process; BPS, bisphenol S; CC, cellular component; FDR, false discover rate; GO, Gene Ontology; KEGG, Kyoto Encyclopedia of Genes and Genomes; MF, molecular function.

Using the DAVID webtool (version 2023q2) and confining the species to *Homo sapiens*, we conducted a KEGG pathway enrichment analysis to determine the roles of the potential targets in specific signaling pathways (Figure 2A). Additionally, we conducted a GO analysis for 42 potential targets. Our extensive examination revealed 1843 significantly enriched GO items in total, indicating the involvement of 1748 BPs, 21 CCs, and 74 MFs. We ranked these GO terms on the basis of their FDR values and selected the top 10 terms in each category (BP, CC, and MF) that presented the lowest FDR values. This allowed us to create a visual representation of the enrichment analysis (Figure 2B). Meanwhile, we mapped the GO assay loop to identify significant biological processes, cellular components, and molecular functions (Figure 2C).

The results of the KEGG and GO analyses revealed that the genes were widely distributed and involved in different biological processes. Many of these genes are essential for important regulatory functions, such as reacting to oxygen, cellular secretion, and metabolic processes. Among the numerous enriched KEGG pathways, some were strongly enriched, such as TNFα, FoxO, estrogen, and IL‐17 pathways.

### 3.5 Molecular Docking of BPS With Core Target Proteins Involved in Reproductive Injury

**FIGURE 3:**
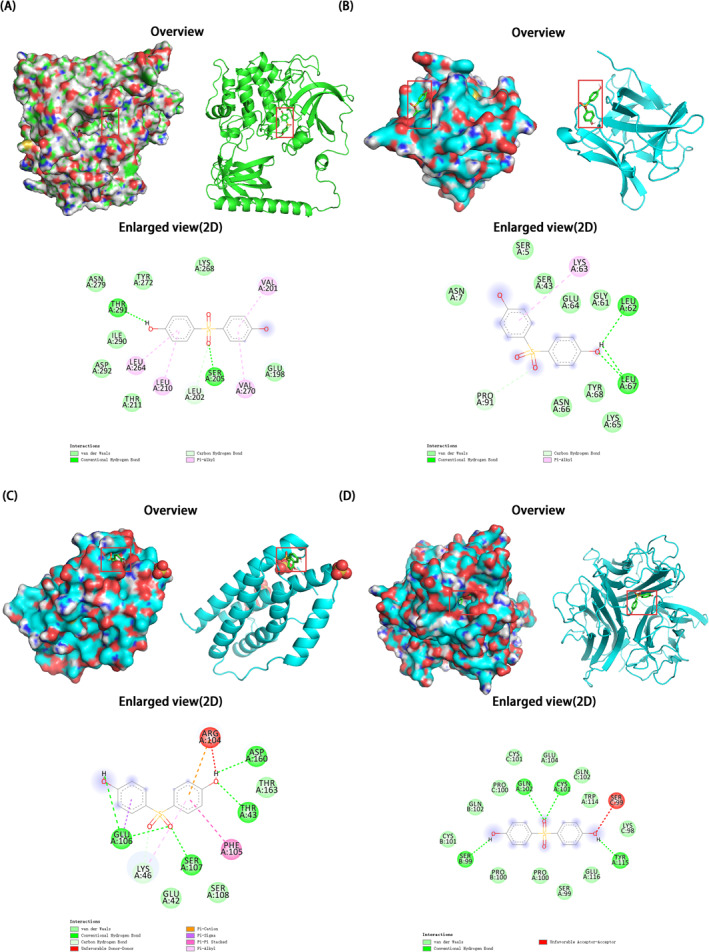
Molecular docking results show that BPS has the lowest binding energy when interacting with the target protein. (A) BPS and AKT1, (B) BPS and IL‐1β, (C) BPS and IL‐6, and (D) BPS and TNFα. AKT1, AKT serine/threonine kinase 1; BPS, bisphenol S; IL‐1β, interleukin‐1‐beta; IL‐6, interleukin‐6; TNFα, tumor necrosis factor α.

To investigate the interactions between BPS and four essential target genes (AKT1, IL‐1β, IL‐6, and TNFα), we employed molecular docking analysis. According to the literature, TNFα, a member of the TNFα family, plays a role in reproductive function, prompting us to select TNFα for molecular docking. The results generated by AutoDock software for the four targets indicated low binding energies, suggesting a strong affinity between BPS and the targets. The strong binding observed between BPS and all four core target proteins is crucial for comprehending the molecular pathways involved in reproductive toxicity and injury caused by BPS. The binding energies were found to be below zero, indicating spontaneous interactions between BPS and the core target proteins. To visualize the minimum energy of binding between each target protein and BPS, a graphical representation was generated via Discovery Studio and PyMOL (Figure 3).

### 3.6 Molecular Docking of BPS With AKT1

**FIGURE 4:**
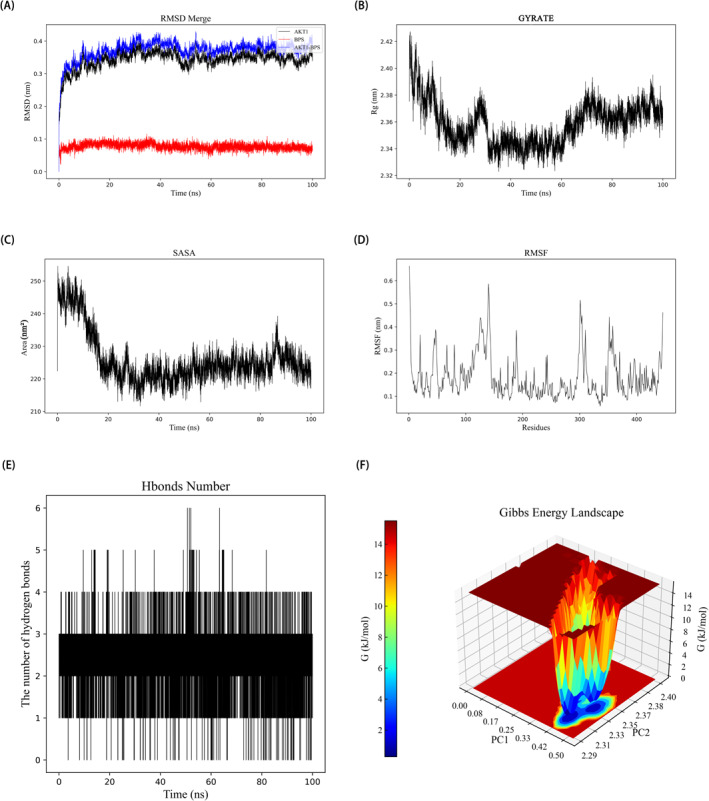
Molecular dynamics of BPS with AKT1. (A) The binding of BPS and AKT1 can maintain stable state. (B) The stable Rg values over time suggest that the complex of BPS and AKT1 maintains a consistent tertiary structure, with minimal expansion or contraction, implying structural stability. (C) The observed stability in SASA values, with minor fluctuations, suggests that the complex's solvent exposure remains relatively constant. (D) The RMSF plot highlights the flexibility of residues within AKT1. Peaks around residues 140, 300 and 350 suggest regions with high flexibility, potentially loops or active‐site‐adjacent areas involved in substrate binding. The increased flexibility in these areas might facilitate ligand interaction, contributing to functional adaptability. Lower RMSF values in other regions indicate rigid, structurally stable segments within AKT1, which maintain the integrity of the protein structure in the complex. (E) There exists a transient yet persistent interaction pattern between AKT1 and BPS. Hydrogen bonds are pivotal in sustaining the integrity of the BPS‐AKT1 complex, significantly influencing both the binding affinity and specificity. (F) The blue and green regions represent low‐energy conformational states, indicating their stability as structures. In contrast, the red regions denote high‐energy states. The presence of a dense low‐energy basin phenomenon suggests that during the simulation process, the BPS‐AKT1 complex adopts one or several predominant conformations, which implies that the binding mode of the complex remains relatively stable within the sampled conformational space. RMSD, root‐mean‐square deviation. SASA, solvent‐accessible surface area. RMSF, root‐mean‐sauare fluctuation. BPS, bisphenol S. AKT1, AKT serine/threonine kinase 1.

To investigate the binding interactions between BPS and AKT1, we conducted molecular dynamics testing. We utilized the RMSD, GYRATE, and SASA to assess the protein structure, overall compactness, and surface area following the binding of small molecules. After AKT1 associated with BPS, the RMSD stabilized within a narrow range, whereas both the GYRATE and SASA values decreased (Figure 4A–C). This finding suggests that the interactions between BPS and AKT1 lead to increased stability and compactness of the protein molecules, accompanied by a reduction in protein surface area. Additionally, the RMSF was employed as an index to evaluate protein dynamics. The RMSF values revealed that the protein exhibited lower fluctuations in the bound regions and greater fluctuations in the unbound regions, indicating that the binding of BPS significantly influences the stability of the protein (Figure 4D). HBond is a metric used to evaluate hydrogen bonds between proteins and small molecules. During the simulation process, numerous hydrogen bonds were formed between BPS and AKT1, particularly those involving key residues in the proteins and significant functional groups in the small molecules (Figure 4E). To further investigate the stability and interaction mechanisms of the small molecule‐protein receptor docking complex, we conducted molecular dynamics simulations of BPS and AKT1 (Figure 4F). We plotted a three‐dimensional free energy landscape using the principal component analysis (PCA)‐derived principal component 1 (PC1) and principal component 2 (PC2) of the RMSD and Rg values of the small molecule‐protein receptor docking complex as the *x*‐ and *y*‐axes, with the Gibbs relative free energy represented as the *z*‐axis. When BPS docks with the protein receptor, its corresponding PC1 value is approximately 4.2. This value, in conjunction with the RMSD curve of the complex, further confirms the exceptional stability of the small molecule‐protein receptor complex.

## 4 Discussion

BPS and other bisphenol compounds have become prevalent as alternatives to BPA due to concerns over BPA's endocrine‐disrupting effects. However, recent studies indicate that BPS and its derivatives may exhibit similar adverse health impacts, including reproductive toxicity and cytotoxicity. These compounds are widely detected in environmental and biological samples, suggesting significant exposure potential. Given these findings, further research is needed to fully understand their toxicological profiles and to inform appropriate regulatory measures.

In this study, we screened 45 potential targets associated with BPS‐mediated reproductive damage through databases including GeneCards, OMIM, the PharmGKB, and the TTD. Using the STRING platform and Cytoscape, a PPI network of these potential targets was constructed and thereby identified four key nodes as core targets involved in BPS‐mediated reproductive toxicity: IL‐6, IL1‐β, TNFα, and AKT1. These key targets have shown to impair reproductive function by regulating inflammation, immune responses, hormone levels, and signal transduction [31, 32].

IL‐6 and IL1‐β are pivotal regulators of the immune system, particularly in the modulation of inflammatory responses. Within the reproductive system, deficiency in IL‐6 and IL‐1β results in enhanced androgen production due to increased SOCS3 and impaired steroidogenesis in the testes, respectively [33, 34]. Additionally, IL‐6 can stimulate pancreatic islet cells to secrete insulin which is linked to the hyperinsulinemia and insulin resistance commonly observed in patients with polycystic ovary syndrome (PCOS) [35]. IL‐1β was demonstrated to induce ovarian granulosa cell apoptosis and a decrease in estradiol synthesis by activating the NF‐κB pathway [36]. The studies suggest that the two cytokines have varying impacts on male and female reproductive functions, primarily through inflammatory processes.

As a multifunctional cytokine, TNFα impacts the immune microenvironment of reproductive tissues through the activation of immune cells. TNFα leads to spermatogenesis dysfunction through BTB disruption, germ cell apoptosis, and suppression of testosterone synthesis [37, 38, 39]. Additionally, TNFα also can impair ovary function through inducing apoptosis [40, 41]. Furthermore, TNFα can induce the production of other inflammatory cytokines, such as IL‐6 and IL‐1β [42, 43], thereby exacerbating germ cell damage and adversely affecting reproductive function.

AKT1 is a protein kinase whose activation typically involves the activation of phosphatidylinositol 3‐kinase (PI3K), and PI3K/AKT signaling pathway can facilitate expression of pro‐inflammation cytokines including IL‐6, IL‐1β, and TNFα [44]. Moreover, the activation of PI3K/AKT results in abnormal male and female reproductive function [45, 46, 47, 48]. Therefore, we focus on AKT1 to perform further research. We conducted molecular docking and dynamic simulations on BPS and AKT1. The molecular docking results indicated that the binding energy between BPS and AKT1 was negative, suggesting that BPS can spontaneously bind to the protein. Additionally, the dynamic simulation experiments indicated that the conformation of the complex remained stable after BPS bound to AKT1. This stability is crucial for biomedical research as it implies that the complex can maintain its structure under physiological conditions and may have a significant impact on the body.

Functional and pathway enrichment analyses of key targets were performed using the DAVID and FUMA webtools. The KEGG pathway enrichment analysis results revealed that four pathways were significantly linked to reproductive damage: TNFα pathway, FoxO pathway, estrogen pathway, and IL‐17 pathway. These findings suggest that BPS notably impacts the proliferation, development, maturation, and apoptosis of reproductive cells, thereby disturbing normal ovarian and testicular functions.

The results of the GO analysis suggest that the pathway through which BPS induces reproductive damage may be associated with epithelial cell proliferation, gland development, and smooth muscle cell proliferation. Although BPS exposure may not adversely affect the fundamental characteristics and growth patterns of trophoblast stem cells (TSCs), it does induce apoptosis and alter gene expression related to cell fusion, potentially impacting placental development and pregnancy [49]. Furthermore, BPS exposure may influence sex hormone levels and disrupt the endocrine system, thereby affecting the normal development and function of various glands. Research has demonstrated that BPS exposure can lead to significant alterations in the expression of microRNA (miRNA) and their target genes in the gonads of male adult zebrafish, resulting in reproductive defects [50]. This phenomenon may also be observed in humans. In the reproductive system, smooth muscle cells are present in multiple locations and play crucial roles in reproductive health and function. Although current experimental findings do not directly address the impact of BPS on smooth muscle cell proliferation, it is plausible that, as an environmental endocrine disruptor, BPS may influence the function of smooth muscle cells through a similar mechanism. This possibility represents a promising avenue for future research.

Molecular docking analysis showed that all four essential target proteins demonstrated stable interactions with BPS, exhibiting binding energies under 0 kcal/mol, which underscores their significant involvement in reproductive damage associated with BPS [51].

Traditional toxicological research generally depends on animal models and utilizes pathology and immunology methodologies to explore the mechanisms through which drugs and environmental toxins have their impacts. With the rapid rise in potential environmental contaminants today, there is an urgent need for approaches that produce evidence of comparable quality to that obtained from conventional research models. To evaluate the safety of the diverse chemicals resulting from industrial processes, innovative methods that move away from extensive animal testing and the protracted timelines characteristic of traditional assays are essential. Additionally, the physiological, genetic, and molecular pathway differences between test animals and humans suggest that conventional toxicological strategies might miss key mechanisms responsible for the toxic effects of environmental pollutants. Furthermore, relying solely on single‐outcome measures for validation cannot deliver a thorough understanding of the potential impacts of environmental pollutants on specific human systems or diseases. Consequently, we employed network toxicology to conduct toxicological investigations from multiple perspectives.

By utilizing extensive databases that encompass biological data and genomics, the intricate molecular mechanisms associated with potential toxins can be elucidated swiftly and thoroughly. The field of network toxicology facilitates the discovery of essential genes and their related pathways by drawing on human gene databases, which further clarifies how chemicals influence the various molecular networks within the human body. In addition, molecular docking methods forecast and model complex binding interactions at biological and molecular levels, including down to the molecular and atomic dimensions. Therefore, the application of network toxicology alongside diverse databases and analytical techniques improves the efficiency of toxicological screening, increases predictive accuracy, and speeds up the evaluation of numerous underexplored emerging environmental toxins. However, depending exclusively on computational simulations derived from network analyses may not adequately reflect the comprehensive real‐world responses to these compounds. Consequently, it is optimal for toxicological research to incorporate both network toxicology and traditional methodologies, substantiating core targets and pathways through comprehensive epidemiological research and pertinent animal studies to yield strong evidence for prevention and treatment initiatives.

In conclusion, we explored the primary targets and associated pathways involved in reproductive harm caused by BPS through network toxicology approaches. Notably, research focused on how environmental contaminants influence the proliferation of smooth muscle cells, especially concerning reproductive system impairment, is still insufficient. This void in existing studies offers a chance for comprehensive future investigations in this developing field. Nevertheless, additional laboratory methodologies, including histopathological and immunological tests, are required to further assess the links between reproductive toxicity and BPS.

## 5 Conclusion

This research clarifies the molecular mechanisms through which BPS induces reproductive toxicity and presents an innovative network toxicology strategy for examining the toxicity of environmental pollutants. By synthesizing various databases and software tools, we pinpointed potential targets linked to the toxic impacts of BPS and performed analyses on key targets and enriched pathways, thus supplying strong evidence of damage caused by environmental toxins. This methodology deepens the comprehension of the molecular mechanisms related to toxicity and provides fresh evidence for creating targeted strategies aimed at preventing and addressing toxic harm.

## Author Contributions

**Siyuan Wang:** investigation, visualization, writing – original draft. **Yan Fu:** methodology, software, investigation. **Xu Huang:** visualization, data curation. **Qitong Guo:** visualization, data curation. **Xiangqin Zheng:** data curation, methodology. **Shengde Wu:** validation, methodology. **Lianju Shen:** conceptualization, funding acquisition, project administration, writing – review and editing. **Guanghui Wei:** supervision, writing – review and editing.

## Funding

This study was supported by the National Natural Science Foundation of China (Grant 81801521); the Natural Science Foundation Project of Chongqing, Chongqing Science and Technology Commission (Grant CSTB2023NSCQ‐MSX0102); and the Postgraduate Research Innovation Project of Chongqing (Grant CYS21225).

## Conflicts of Interest

Shengde Wu is a member of *Pediatric Discovery* Editorial Board. To minimize bias, he was excluded from all editorial decision‐making related to the acceptance of this article for publication. The other authors declare no conflicts of interest.

## Data Availability

The data that support the findings of this study are available from the corresponding author upon reasonable request.
